# An ambitious global goal on adaptation for heritage

**DOI:** 10.1038/s44168-026-00379-x

**Published:** 2026-04-22

**Authors:** Nicholas Simpson, Salma Sabour, Luckson Zvobgo, Andrew Potts, Portia Adade Williams, Joanne Clarke, Christopher H. Trisos

**Affiliations:** 1 Climate Risk Lab, African Climate and Development Initiative, University of Cape Town, Cape Town, South Africa; 2 African Synthesis Centre for Climate Change, Environment and Development (ASCEND), University of Cape Town, Cape Town, South Africa; 3 Preserving Legacies, Washington, DC USA; 4 Department of Archaeology, Durham University, Durham, UK; 5 CSIR - Science and Technology Policy Research Institute, Accra, Ghana

**Keywords:** Environmental studies, Social policy

## Abstract

Getting heritage-relevant adaptation indicators right is critical for adapting heritage to climate change. We assess the Belém Adaptation Indicators adopted at COP30 under the UAE Framework for Global Climate Resilience to clarify strengths and gaps. Adoption is a significant step toward operationalising the Global Goal on Adaptation. To close outstanding gaps, we call for clear definitions, baselines and metadata that reflect tangible and intangible heritage, including diverse knowledge systems, while minimising reporting burdens by aligning with UNESCO inventories and national channels. We flag omissions and propose pragmatic fixes for credible, low‑burden monitoring and action including mixed‑methods and narrative ‘direction‑of‑travel’ measures, capacity strengthening and finance access to manage losses and damages and reposition heritage as a driver of climate‑resilient development.

## Introduction

The world’s natural and cultural heritage is increasingly vulnerable to climate change through damage to physical sites, disruption of practices, and erosion of cultural identity, with Indigenous Peoples, local communities and other groups who, in many contexts, experience heightened risk of loss and damage due to intersecting forms of marginalisation. For example, coastal heritage faces escalating exposure to sea‑level rise, flooding and erosion, with documented risks to World Heritage properties^1–4^. Yet heritage systems can be mobilised as enablers of adaptation and support incremental, restorative and transformative pathways through place‑based knowledge, identity and social cohesion.

Within this opportunity space, Parties at COP30 adopted the Belém Adaptation Indicators, including Target 9(g) on cultural heritage, providing a practical entry point to operationalise heritage‑relevant adaptation metrics. The Global Goal on Adaptation (GGA), established under the Paris Agreement and operationalised via the UAE Framework for Global Climate Resilience, aims to enhance adaptive capacity, strengthen resilience, and reduce vulnerability^5^. Operationalising the GGA targets and indicators entails agreeing indicator definitions and metadata, specifying methods and baselines, establishing reporting channels, and initiating periodic reporting^6,7^.

However, operationalizing this goal has proven difficult due to the inherently context-specific and multidimensional nature of adaptation^8^. The Belém Adaptation Indicators proposed, “Protecting cultural heritage from the impacts of climate-related risks by developing adaptive strategies for preserving cultural practices and heritage sites and by designing climate-resilient infrastructure, guided by traditional knowledge, Indigenous Peoples’ knowledge and local knowledge systems” ^5^. Building on the inclusion of cultural heritage, this commentary argues for an ambitious, inclusive, and empirically grounded GGA for Heritage.

### Indicators to measure progress on heritage adaptation

Efforts to measure adaptation progress globally have been complicated by the need to simplify complex realities into quantifiable indicators, and as a result, the GGA has encountered a range of conceptual, technical, and political challenges^9^. These indicators, often composite in nature, risk obscuring local nuances and can rely on proxies that inadequately capture the phenomena they intend to measure^9^. This is particularly challenging in the heritage context, where cultural heritage may include intangible characteristics. Further, cultural heritage remains significantly underrepresented in existing adaptation datasets and indicators with only a single dataset explicitly addressing Indigenous lands, with no comprehensive, climate-specific indicators for cultural heritage^9^.

Beyond technical hurdles, four factors hamper progress. Firstly, political prioritisation that favours sectors with immediate economic metrics subsequently affecting prioritization of heritage in climate finance mechanisms. Secondly, institutional fragmentation separating culture/heritage from climate agencies creating coordination gaps that limits effective mainstreaming of heritage into plans and policy frameworks. Thirdly, methodological biases that privilege tangible assets and standardised datasets over place‑based and relational heritage values limiting such representation in adaptation metrics. Fourthly, the emerging nature of concepts, methods and expectations around assessing and reporting the effectiveness of climate change adaptation in the heritage field, together with the ongoing evolution of existing heritage reporting processes, which, including under the World Heritage Convention’s Periodic Reporting framework, are only beginning to incorporate climate change adaptation and its effectiveness in a more systematic way^10^. These dynamics particularly disadvantage communities whose heritage governance does not map neatly onto Western frameworks. To minimise reporting burdens we prioritise (i) harvesting data from existing heritage reporting (e.g., UNESCO mechanisms and national inventories), while advocating their progressive strengthening for climate adaptation monitoring (ii) integrating heritage fields into ongoing adaptation instruments (such as NAPs, NDCs, BTRs), and (iii) allow use of qualitative reporting where quantitative data are unavailable, using ‘direction‑of‑travel’ and narrative indicators that can be captured through brief, periodic submissions (e.g., community narratives of continuity of practice) with free, prior and informed consent (FPIC). These approaches aim to enhance and repurpose existing systems, maintain inclusivity for diverse heritage knowledge and governance while avoiding the creation of parallel data systems that would add to reporting burdens^9^. Where Parties’ NAPs do not yet contain heritage‑specific indicators, we recommend briefly annotating existing sectoral entries (e.g., water, settlements, ecosystems) to identify heritage‑relevant activities and, where appropriate, appending short community narratives of continuity and change under FPIC rather than creating new standalone heritage templates.

### Toward an Ambitious Global Goal on Adaptation for Heritage

Transformative adaptation requires transformation among those who fund, plan, implement and evaluate interventions^11^. The Heritage Adapts to Climate Alliance (HACA) published in June 2025 a position paper recommending key improvements for integrating cultural heritage into the GGA^12^. Supporting the inclusion of cultural heritage in Target 9 g^5^, HACA seeks to operationalize it through a refined set of indicators that reflect the multidimensional nature of heritage and its role in adaptation (Box 1).

HACA’s proposed subcomponents for Target 9 g that include protection of cultural practices, heritage sites, and climate-resilient infrastructure guided by IK (Indigenous Knowledge) and LK (local knowledge) aim to concretise the GGA’s ambition. For example, communities may contribute brief oral histories of changes in seasonal cues and associated practice adaptations as a narrative indicator of continuity and resilience, complementing any quantitative site‑level measures. While the previous set of GGA indicators relied heavily on percentage-based metrics^9,12^, HACA called for indicators that better capture incremental progress and community-led innovation. It also highlights cross-linkages between cultural heritage and other GGA targets (e.g., food, water, ecosystem and biodiversity, infrastructure and human settlement, impacts, vulnerability and risk, planning and MEL), advocating for biocultural approaches^13^ and co-developed indicators that reflect the interdependence of cultural and ecological systems and thereby greater recognition of the contribution of heritage for adaptation^13–15^.

HACA also highlights the need for robust capacity-building mechanisms tailored to cultural heritage. It critiques the imbalance within previous GGA indicators, noting a lack of outcome and impact indicators. HACA calls for indicators that measure long-term resilience, transmission of knowledge, intergenerational learning, and the empowerment of youth and Indigenous Peoples as cultural knowledge holders. Furthermore, HACA advocates for inclusive and locally led adaptation processes, echoing the UNFCCC’s call for participatory approaches. Indicators such as participation in policy processes (9g47) and training programs for conservation professionals (9g26) are highlighted as essential for building the adaptive capacity of heritage custodians and communities.

HACA further highlights the omission of explicit references to IK and LK in Target 9 g subcomponents. It calls for indicators that measure the integration of these knowledge systems into infrastructure design (e.g., Targets 9g12 and 9g56) and stresses the need for ethical engagement in the use of IK and LK, advocating for indicators that ensure free, prior, and informed consent and the protection of intellectual and cultural rights.

Finally, HACA identifies significant gaps in adaptation finance for cultural heritage and calls for dedicated indicators to track financial flows. It supports indicators such as funding secured for heritage adaptation (9g19) and budget lines for heritage adaptation (9g22), and proposes new indicators to measure the share of adaptation finance allocated to heritage projects led by Indigenous Peoples and local communities. HACA also recommends economic valuation indicators (e.g., total economic value of preserved heritage due to adaptation) to support cost-benefit analyses and justify investment in heritage adaptation.

Alignment with the ‘means of implementation’ of climate action is critical for operationalizing a Global Goal on Adaptation for heritage. This includes finance, capacity-building, and technology transfer^6^. Indicators should not only measure outputs but also track institutional arrangements for training, integration of Indigenous and local knowledge into adaptation technologies, and equitable access to finance. Dedicated heritage finance indicators, such as budget lines and funding flows for community-led projects, can address current gaps and ensure predictable resources. Similarly, capacity-building must prioritize heritage professionals and custodians through sustained training programs, while technology mechanisms should support context-sensitive innovations like climate-resilient retrofitting and digitization. Embedding these elements within NAPs, NDCs, and biennial transparency reports could strengthen coherence across adaptation planning and ensure heritage is recognized as both vulnerable and vital for climate resilience^16^.

Box 1 To realize the full potential of the Global Goal on Adaptation for heritage, the HACA position recommends we need to do the following

1. Move beyond risk avoidance to embrace heritage as a catalyst for thriving futures. Qualitative and narrative-based methods can complement quantitative indicators in capturing the dynamics of heritage adaptation.
2. Use direction-of-travel metrics, disaggregate by gender, age, and Indigeneity, and ensure indicators reflect ethical engagement and community agency.
3. Integrate heritage into National and Global Planning and ensure heritage is included in NAPs, NDCs, other Adaptation Strategies and Frameworks and climate finance mechanisms.
4. Heritage adaptation also can benefit from alignment with broader international frameworks, such as the Sustainable Development Goals and the Sendai Framework for Disaster Risk Reduction, which emphasize the protection of cultural assets and the role of culture in resilience-building.
5. Avoid maladaptation and screen adaptation strategies for unintended consequences.
6. Prioritize co-developed, culturally appropriate responses that enhance rather than erode heritage values.
7. Support training for heritage professionals, fund community-led adaptation, and promote intergenerational knowledge transmission.

### The Process of Indicator Development and Adoption

Parties at COP30 adopted the Belém Adaptation Indicators, including Target 9(g) on cultural heritage, as voluntary, non‑prescriptive anchors for national reporting. Relative to the technical experts’ proposed list, however, the adopted 9(g) set omits an explicit indicator on climate‑resilient infrastructure/retrofitting of heritage buildings. This gap that risks under‑protecting substantial historic building stocks if not addressed through other reporting channels. To implement 9(g) while avoiding parallel systems, Parties can define “at‑risk heritage” within the national risk and vulnerability assessments referenced in NAPs and BTRs, drawing on UNESCO inventories and national or local registries as inputs. Where heritage‑relevant retrofitting is undertaken, progress can be reflected via cross‑target reporting under infrastructure and human settlements (e.g., Target 9(e)), with clear metadata for terms such as “establishment” (of institutional arrangements) and “sustained engagement” (with Indigenous Peoples and/or local communities) to reduce tokenistic reporting. Table 1 therefore compares the experts’ proposed indicators with the adopted set and offers illustrative outcome measures that Parties could pilot alongside the adopted metrics (e.g., percentage of heritage places sustaining community use post‑adaptation; proportion of intangible practices reporting continuity or revival over a defined period). This approach preserves the voluntary character of the Belém framework while enabling consistent, low‑burden accounting for heritage adaptation.

**Table 1:** Comparing the proposed indicators by technical experts and adopted indicators as provided by the COP 30 Presidency for GGA Target 9g on Cultural heritage

Proposed indicators by Technical Experts	Adopted indicators from the COP Presidency (COP30, Belém, November 2025)
9g01: Percentage of at-risk cultural and natural heritage sites with adaptation measures implemented 9g02: Percentage of intangible cultural heritage elements with enhanced resilience to climate change impacts	(a) Percentage of at-risk cultural and natural heritage sites and elements with adaptation measures implemented to enhance resilience to climate-related hazards under different warming scenarios, as appropriate for regions and contexts, guided by traditional, local or Indigenous Peoples’ knowledge and practices, disaggregated, as appropriate, by tangible and intangible cultural elements;
9g03: Proportion of cultural heritage protected from climate impacts by (i) digitizing for preservation and recovery, (ii) storing movable heritage in climate-resilient facilities	(b) Proportion of cultural heritage protected from climate impacts through digitization measures for preservation and recovery and by storing movable heritage in climate-resilient facilities;
9g04: Percentage of cultural heritage with emergency preparedness and response plans in place for climate change related hazards	(c) Percentage of cultural heritage and sites with adaptation measures and emergency preparedness plans in place for climate change related hazards under different warming scenarios, as appropriate for regions and contexts;
9g05: Percentage of climate change adaptation plans, policies and strategies that incorporate the safeguarding and protection of cultural heritage 9g07: Percentage of cultural heritage specific climate adaptation measures that engage with and are informed by local or Indigenous Peoples and their knowledge systems	(e) Percentage of climate adaptation measures focused on cultural heritage that maintain sustained engagement with Indigenous Peoples and/or local communities.
9g06: Number of relevant climate change adaptation training programmes that integrate cultural heritage and/or guidance from traditional, local or Indigenous knowledge.	(d) Level of establishment of institutional arrangements for the provision of regular training on climate change adaptation that incorporates guidance from traditional, local, and Indigenous Peoples’ knowledge where applicable;
9g08: Number of cultural heritage buildings and sites retrofitted with climate-resilient materials and/or technologies, including those guided by traditional, local, or Indigenous building practices	—

Target Language: “Protecting cultural heritage from the impacts of climate-related risks by developing adaptive strategies for preserving cultural practices and heritage sites and by designing climate-resilient infrastructure, guided by traditional knowledge, Indigenous Peoples’ knowledge and local knowledge systems”.

Although efforts were made to retain most of the indicators from the proposed indicators from technical experts for cultural heritage with minimal changes to the new list from the COP30 Presidency, it is important to note that some key aspects of the target components were lost completely. For example, protecting cultural heritage by designing climate-resilient infrastructure was lost completely in the new list. Excluding this dimension risks under‑protecting extensive stocks of historic buildings worldwide that require adaptation through context‑sensitive retrofits. We therefore flag this gap for future technical work and note feasible proxies under infrastructure and human settlements targets (e.g., 9e), which Parties could cross‑reference in heritage‑relevant reporting.

The consolidation from eight proposed indicators to five adopted indicators represents an important advance for global adaptation governance. It signals a clearer recognition of culture and heritage within an operational adaptation measurement framework under the UNFCCC. Adaptation measures must now, where possible, be guided by traditional, local, and Indigenous practices; institutional training arrangements are expected to incorporate these knowledge systems; and implementation should where possible, include sustained engagement with Indigenous Peoples and local communities. Taken together, this has the potential to shift practice away from instrumental consultation towards the recognition of diverse knowledge systems as integral to effective adaptation, in line with IPCC and UNFCCC norms that position Indigenous Peoples and local communities as essential partners in effective adaptation. At the same time, several of the adopted formulations raise questions about how they will be operationalised. In particular, the indicator on the percentage of heritage adaptation measures that maintain ‘sustained engagement’ with Indigenous Peoples and/or local communities is likely to function more as a normative benchmark than as a fully trackable metric in many real‑world contexts, especially where formal stewardship rights are limited or data systems do not yet capture the quality and continuity of engagement. Its value may therefore lie both in measuring progress where such partnerships already exist and in exposing governance gaps where they do not, provided it is accompanied by complementary, more immediately implementable approaches. One significant evolution is the elevation of traditional, local or Indigenous Peoples’ knowledge from a peripheral component to a cross-cutting foundation (across 60% of the adopted indicators). The framework also moves from activity-based to systems-based measurement. For example, the earlier output metric that simply counted the number of climate adaptation training programmes (9g06) has been replaced by an indicator that assesses the establishment of institutional arrangements for regular adaptation training, which better captures whether enduring capacity exists within universities, heritage agencies and governance structures, including through long‑term partnerships with NGOs, community organisations and specialist private providers. Additional improvements include scenario-based planning across current and future Global Warming Levels linked to goals in the Paris Agreement, explicit integration of both tangible and intangible heritage, and clearer separation between planning and implementation functions.

The adopted indicator architecture is also more complex to operationalize, which could undermine implementation and substantial measurement and capacity challenges, especially in low- and middle-income countries. The adopted indicators only partly reconcile the need for measurability with the complexity of cultural heritage. Percentage-based metrics risk oversimplifying relational and place-based heritage realities, particularly for informal, undocumented, or non-listed heritage. Taken together, the adopted indicators represent an important step towards integrating culture and heritage into adaptation measurement under the UNFCCC. To support consistent reporting, Parties could define “at‑risk heritage” within national risk and vulnerability assessments referenced in NAPs or BTRs, drawing on UNESCO inventories and local registries for inputs. Clear metadata on terms such as “establishment” of institutional arrangements (indicator d) and “sustained engagement” (indicator e) will be essential to avoid tokenistic reporting. Structural imbalances in the Monitoring, Evaluation, and Learning (MEL) framework compound these challenges. Indicators (a), (b), and (c) capture implementation outputs and outcomes, while only indicators (d) and (e) address input and process dimensions, and none measure adaptation effectiveness or long-term heritage resilience. The framework, therefore, captures what is most easily quantified but risks overlooking relational, intergenerational, and transformative aspects of heritage. Institutional capacity constraints remain significant, coordination between heritage and climate sectors is often weak, and financial resources are insufficient to support meaningful implementation. Without strong verification and accountability mechanisms, the risk of superficial and tokenistic reporting persists. Most importantly, the burden of implementation falls disproportionately on LDCs, LLDCs, and SIDS, countries with the least resources but the highest vulnerability, raising critical questions of equity in global adaptation governance. To rebalance process- and outcome-oriented metrics, Parties could, where feasible, pilot outcome‑oriented measures, such as: (i) percentage of heritage places that sustain community use or access after adaptation interventions; and (ii) proportion of intangible heritage practices that report continuity or revival following adaptation support over a defined period. Such cross‑target linkages have concrete implications for planning and budgetary. Wherever heritage materially contributes to outcomes in water, food systems, ecosystems, health, or settlements, Parties should reflect this explicitly in sectoral plans and budgets, using cross‑referenced indicators and simple budget tags to track the allocation of resources to heritage-relevant adaptation. Beyond the Target 9(g) indicators, Supplementary Table 2 shows that 28% of the original 100 proposed indicators from technical experts already intersected with ecosystem resilience (9 d), infrastructure (9e), and livelihoods (9 f), demonstrating that heritage concerns were never confined to Target 9(g) alone but were inherently cross-cutting across the adaptation framework. Building on this foundation, Supplementary Table 3 shows that this cross-sectoral relevance becomes even more pronounced in the adopted Belém indicators: 32 of the 59 Belém indicators (54%) have direct or indirect relevance to cultural heritage, underscoring heritage’s deeply cross-sectoral importance and role in adaptation. Indicators on ecosystem-based adaptation and nature-based solutions (9 d(f)), settlement upgrading with local engagement (9e(a)), and heritage-based livelihoods within social protection systems (9 f(a–c)) could effectively reflect heritage’s contributions to community resilience and wellbeing. Indicators on water basin adaptation (9a(d)), agricultural practices (9b(a)), health support (9c(d)), and institutional knowledge frameworks (9b(b)) further intersect with heritage concerns. This would align with recent progress across climate and biodiversity assessments that have recognised that heritage includes cross-cutting sectoral boundaries, such as terrestrial and freshwater ecosystems, oceans and coastal ecosystems, economies, poverty and livelihoods. Nevertheless, the current framing of Target 9(g), while a vital starting point, remains narrowly focused on cultural heritage and risks excluding natural heritage and hybrid forms of heritage that are deeply embedded in community identity and ecological stewardship. A more integrative framing would better reflect the full adaptive potential of heritage across interconnected social–ecological systems.

### Reframing Heritage Adaptation

Traditional adaptation frameworks often emphasize risk avoidance^17^. While necessary, a risk‑centric view can overshadow the diverse roles of heritage in adaptation, including but not limited to its transformative potential in sustaining identity, social cohesion and human–nature reciprocity. Heritage‑based adaptation can include community guardianship models and ecosystem restoration of culturally significant landscapes, linking continuity of practice with ecological resilience. It also underpins more incremental and protective forms of adaptation, for example by informing climate‑sensitive retrofits of historic buildings, guiding land‑use decisions that avoid maladaptation, and sustaining everyday practices that reduce risk without requiring fundamental social or spatial transformation.

Heritage supports adaptation by fostering identity and embedding local and Indigenous knowledge systems into adaptation strategies. It strengthens the cultural relevance and legitimacy of adaptation strategies and opens space for people to define what a “liveable” future means in their own terms, contributing to a shift toward an aspirational model of adaptation that fosters reciprocal relationships between people and nature, and acknowledges the realities of adaptation limits, including how to manage losses and damages. This ‘reweaving’ of human–nature relationships through heritage could support futures grounded in justice, equity, care, meaning, and belonging and plays key roles in nature-based solutions (e.g., mangrove restoration, traditional land management), cultural continuity (e.g., storytelling, rituals), climate change literacy (e.g., museums, education), and livelihood diversification (e.g., cultural tourism, crafts)^1,18–24^. In this sense, heritage functions simultaneously as a resource for incremental, restorative and transformative adaptation pathways. We recognise also that emphasising heritage can surface intra‑community debates about which traditions are prioritised. Transparent, inclusive processes with FPIC and attention to intergenerational equity are therefore essential.

At COP30, Parties adopted the Belém Adaptation Indicators under the UAE Framework for Global Climate Resilience, marking an important step towards operationalizing the Global Goal on Adaptation^25^. For cultural heritage (Target 9 g), these indicators include measures such as the percentage of at-risk heritage sites with adaptation actions, resilience of intangible heritage, digitization for preservation, emergency preparedness, integration into adaptation plans, training programs incorporating Indigenous knowledge, and retrofitting of heritage buildings. These voluntary, non-prescriptive indicators aim to inform national adaptation planning and reporting processes, including NAPs, NDCs, and biennial transparency reports, while respecting national circumstances and avoiding additional reporting burdens. Their adoption signals recognition of heritage as both vulnerable and vital for climate resilience, and underscores the need for dedicated finance and capacity strengthening to implement heritage adaptation at scale. They also create, for the first time in the UNFCCC context, an explicit anchor through which heritage can be reflected in cross‑sectoral adaptation monitoring and finance debates.

The current GGA heritage indicators in the Belém Adaptation Indicators align with several HACA principles, particularly in the mainstreaming of inclusion, capacity-building, and the recognition of Indigenous knowledge. Yet, they remain heavily weighted toward tangible outputs, institutional uptake, and technical interventions, risking the neglect of ethical engagement, plural knowledge systems, and cultural meaning central to transformative adaptation. Prioritizing what is readily measurable may sideline more complex, context-dependent dimensions of heritage resilience that resist quantification or standardization. The framework, therefore, captures what is most easily quantified but risks overlooking relational, intergenerational and transformative aspects of heritage, as well as more incremental, everyday practices that sustain cultural continuity under changing climatic conditions. During synthesis of the available information for target 9 g, it is critical for that process to address some of the imbalances and short falls in the current list of indicators.

Moreover, without explicit cross-referencing between thematic and implementation targets, and without finance indicators tracking heritage-related support, heritage risks exclusion from both policy and funding. The architecture of adaptation finance under the GGA presents political and technical barriers to the inclusion of heritage. Politically, the UAE Framework separates thematic targets (e.g., Target 9 g on heritage) from implementation targets, with only the latter linked to finance, capacity-building, and technology transfer. This structural divide leaves heritage without a direct pathway to dedicated funding. Technically, finance indicators are concentrated under Target 10c, which supports the development of National Adaptation Plans (NAPs). These plans often prioritize sectors like water, agriculture, and infrastructure, sidelining heritage. The voluntary nature of reporting and the complexity of indicators further challenge inclusion, especially for LMICs with limited capacity. Without clear guidance on how to reflect heritage contributions within existing sectoral entries, for example, in water, food systems, health or settlements, heritage risks remaining invisible in budget lines even where it substantively supports adaptation outcomes.

Given these constraints, an ambitious yet pragmatic GGA for Heritage must advocate for inclusive, context-sensitive indicator frameworks and financing mechanisms that make heritage adaptation measurable, fundable, and actionable. This implies working with a pragmatic mix of quantitative, qualitative and narrative indicators that can be implemented under real‑world capacity constraints, while still incentivising Parties to progressively deepen how they account for heritage‑related adaptation.

The UNFCCC or global heritage bodies such as UNESCO could develop guiding frameworks that support context-sensitive, co-developed indicator systems. These should complement GGA quantitative metrics with qualitative evaluations where feasible, recognising that global frameworks must balance comprehensiveness with standardization requirements. Refining heritage indicators to better capture community-driven, participatory, and cross-sectoral dimensions is essential for shifting global adaptation monitoring from viewing heritage as only vulnerable and at risk from climate change to recognizing it as a dynamic agent in climate resilience - supporting incremental risk reduction, enabling social and ecological recovery after climate shocks, and, where appropriate, contributing to deeper transformative change - by advancing justice, sustaining cultural continuity, and contributing meaningfully to transformative adaptation.

This commentary synthesises publicly available UNFCCC documentation on the GGA and the Belém Adaptation Indicators and draws on selected published literature. It does not provide an empirical evaluation of indicator implementation within Parties, nor does it test reporting feasibility across different administrative contexts. Data limitations persist for intangible and non‑listed heritage, and there are risks of inconsistency or tokenism where metadata remain under‑specified. Our proposals aim to minimise reporting burdens by aligning with existing heritage and adaptation mechanisms, but feasibility will vary with institutional capacity and resources. These limitations should be considered when interpreting the scope and generalisability of our recommendations.

## Supplementary information

Supplemental Material.

## Data Availability

No datasets were generated or analysed during the current study.
