# Supplementary material for: An ambitious global goal on adaptation for heritage
Source: NPJ Clim Action. 2026 Apr 22;5(1):50. doi: 10.1038/s44168-026-00379-x (PMC13099379; doi:10.1038/s44168-026-00379-x)
Supplement: Supplementary file 1 — Supplemental Material. [file 44168_2026_379_MOESM1_ESM.docx]

Supplemental Material

**An ambitious Global Goal on Adaptation for Heritage**

Table of Contents

[1 Supplemental Table 1: Description, rationale, data availability and unit of measurement of the list of potential indicators for GGA Target 9g Cultural heritage 1](#_Toc215847574)

[2 Supplemental Table 2: List of potential indicators under other GGA targets determined to have relevance to GGA Target 9g Cultural heritage 7](#_Toc215847575)

[3 Supplemental Table 3: List of potential adopted indicators under other GGA targets determined to have relevance to GGA Target 9g Cultural heritage 9](#_Toc215847576)

# **Supplemental Table 1: Description, rationale, data availability and unit of measurement of the list of potential indicators for GGA Target 9g Cultural heritage**

| **INDICATOR ID** | **DESCRIPTION** | **RATIONALE** | **DATA AVAILABILITY** | **UNIT OF MEASUREMENT** |
| --- | --- | --- | --- | --- |
| Target 9(g) Protecting cultural heritage from the impacts of climate-related risks by developing adaptive strategies for preserving cultural practices and heritage sites and by designing climate-resilient infrastructure, guided by traditional knowledge, Indigenous Peoples’ knowledge and local knowledge systems | | | | |
| 9g01: Percentage of at-risk cultural and natural heritage sites with adaptation measures implemented | This indicator measures the level of implementation of adaptation measures on cultural and natural heritage sites identified as at risk. This includes adaptation measures implemented for different types of cultural and natural heritage lists (international listed and listed by Parties - Parties have national, subnational, and local lists of classified/designated heritage sites) and non-listed sites.  Cultural and natural heritage sites at risk from climate impacts are identified through national and subnational (local) or sectoral impacts, risk and vulnerability and other relevant assessments.  Implemented measures include existing adaptive strategies that are supported through laws, regulations, or community support, as well as new adaptation measures implemented. | Cultural and natural heritage is increasingly exposed to climate change hazards and impacts, placing them increasingly at-risk of impacts that threaten their existence, their cultural values, and their continued access and use. Therefore, it is critical for cultural and natural heritage sites to be protected from increasing climate change impacts through the implementation of site specific adaptation measures and actions. Such adaptation measures may include new and innovative solutions using new technologies or simply ensuring support for the continuation of long-standing local adaptation measures that have enabled the heritage sites to be responsive to its geography and survive through generations. It is critical to also include non-listed sites as many of these are not documented or classified under formal systems.  The IPCC AR6 assesses with medium confidence that ""cultural losses, related to tangible and intangible heritage, may result in irrevocable losses of sense of belonging, valued cultural practices, identity and home, particularly for Indigenous Peoples."" " | Lists of cultural and natural heritage sites are available with Parties at different levels of government. List of at-risk sites could be developed via NAPs or other national documents (NDC, ADCOMs, BTR national communication, etc.), other sources where such assessments exist or can be undertaken such as IPCC, academic literature, grey literature eg implementation reports, other organisations and networks including heritage adaptation professionals networks; UNESCO, data is also available from whc.unesco.org/en/indicators2030/ and https://whc.unesco.org/en/list/ and https://www.unesco.org/en/iggp/geoparks/about  and https://www.ramsar.org/document/list-wetlands-international-importance-ramsar-list and https://unesdoc.unesco.org/ark:/48223/pf0000394251 | Percentage |
| 9g02: Percentage of intangible cultural heritage elements with enhanced resilience to climate change impacts | This indicator measures the extent to which adaptation measures and actions that are implemented have enhanced the resilience of communities to develop the capacity to adapt to climate related adversity to safeguard and continue practicing their intangible cultural heritage. Resilience can be developed through the support for laws, regulations and measures put in place by the state party at various levels of government or at an international level. Intangible cultural heritage elements, including practices and knowledges that continue to thrive demonstrate enhanced resilience in the face of climate change impacts. | This indicator focuses on ensuring that intangible cultural heritage elements (festivals, ceremonies, oral traditions, practices, and knowledges etc.) continue to thrive in communities where they are most meaningful, demonstrating that adaptation have been successfully implemented in response to climate risks to ensure their resilience. It also provides a global picture of the extent to which intangible practices have been made more resilient through climate adaptation. Cultural practices are dynamic and often not included in formal inventories. | "https://whc.unesco.org/en/culture2030indicators/  Internationally listed intangible cultural heritage elements by UNESCO is found here: https://ich.unesco.org/en/ and whc.unesco.org/en/indicators2030/; NAPs or other national documents (NDC, ADCOMs, BTR national communication, etc.). Academic literature, NGO reports, and other sources can provide additional data, including diverse climate adaptation heritage professional networks outside of UN agencies " | Percentage |
| 9g03: Proportion of cultural heritage protected from climate impacts by (i) digitizing for preservation and recovery, (ii) storing movable heritage in climate-resilient facilities | The cultural heritage protected includes all forms of climate vulnerable tangible heritage (built, archaeological, movable, and underwater etc.), natural heritage, and intangible cultural heritage. Listed cultural heritage includes listed (at international level, listed by Parties, national, subnational and local level lists). Parties have national, subnational, and local lists of classified/designated cultural heritage and other heritage that might not be listed. Specific focus on knowledge continuity of at-risk heritage collections; the indicator focuses on the percentage of important cultural items that are in danger from climate change. This includes focus on use of technology to preserve cultural heritage as well as to document them in a form that supports recovery of cultural heritage that might be threatened by climate change impacts. | Use of new technologies to document and preserve cultural heritage is an important way to protect knowledge about them for future generations as well as for recovery and reconstruction of cultural heritage when affected by climate change impacts and hazards. Digitization of cultural heritage elements will ensure that in the event of climate -related hazards, such as floods, fire, at least documentation on them will be protected. Climate resilient structures are constructed to store the most valuable movable cultural heritage. Storing movable heritage in climate-resilient facilities protects cultural heritage from climate-related impacts including hazards as well as slow onset events. | "Data on cultural and natural heritage lists available through international organizations and Parties lists at various level of government as well as private museums (for example UNESCO Culture Sector) Data on digitazation is available through a variety of international organizations including the EU, UNESCO, CBD, UNEP, ICOM, IUCN etc. Data is needed on specifically climate vulnerable cultural heritage, both listed and non-listed. https://whc.unesco.org/en/indicators2030/ ; and https://whc.unesco.org/en/; and  https://unesco.org/en/emergencies/culture/ " | Percentage |
| 9g04: Percentage of cultural heritage with emergency preparedness and response plans in place for climate change related hazards | This indicator measures the existence of emergency preparedness and response plans for those cultural heritage sites, objects and elements/practices that are recognized, protected and safeguarded by the Parties (international, national, subnational, and local levels), and their implementation. "In place" means that Parties have not only proposed such measures in the management and safeguarding plans, but that these "emergency preparedness and response plans" have also been implemented and operationalized to avert or minimize impacts in the event of a climate-related hazards. Thus, the indicator focuses on two things (i) developing emergency preparedness and response plans for relevant sites/practices; (ii) the proposed actions have been operationalized and preparedness measures as well as response readiness measures are in place. Parties could report on these two levels. Details to be included in the metadata. | Climate change related events and hazards, increasingly common in recent years, are having devastating impacts on cultural heritage sites and elements/practices. Having emergency preparedness and response plans focused on climate related hazards allows Parties to have a detailed procedure on preparedness measures to avert and minimize impacts and response measures for actions in case of climate change related hazards. This includes both slow onset and rapid onset events. | Some data is available with UNESCO for cultural and natural heritage sites (including World Heritage sites, Biosphere reserves, and Geoparks); as well as for intangible cultural heritage elements. https://www.unesco.org/en/links and https://whc.unesco.org/en/ and https://ich.unesco.org/en/ and Safeguarding Culture in Times of Crisis \| UNESCO and whc.unesco.org/en/indicators2030/; Under the UNFCCC Convention, data is potentially available through NAPs or other national documents (NDC, ADCOMs, BTR national communication, etc.), other sources eg IPCC, academic literature, grey literature eg implementation reports | Percentage |
| 9g05: Percentage of climate change adaptation plans, policies and strategies that incorporate the safeguarding and protection of cultural heritage | This indicator measures the extent to which adaptation plans, policies and strategies at national or subnational levels include adaptation measures for cultural heritage (tangible, intangible cultural heritage, and natural heritage) to reduce cultural heritage vulnerability to climate change as well as climate change impacts on cultural heritage. | National, sub-national and local climate change adaptation plans, policies, and strategies, often exclude considerations of cultural heritage as a standalone sector compared to other sectors/themes such as agriculture, water, health, infrastructure, ecosystems. Therefore, this indicator aims to streamline and mobilize Parties to integrate considerations of cultural heritage and increase the prominence of cultural heritage in adaptation planning and implementation. This can increase the implementation of adaptation measures that protects and safeguard various forms of cultural heritage globally. It can give a global picture of cultural heritage integration to climate adaptation, with optimal value of all countries integrating cultural heritage into adaptation with focus on climate vulnerability | Some data is available with UNESCO  whc.unesco.org/en/indicators2030/ and from Parties from authorities responsible for developing adaptation plans at national, subnational, and local levels; NAPs or other national documents (NDC, ADCOMs, BTR national communication, etc.), other sources eg IPCC, academic literature, grey literature eg implementation reports | Percentage |
| 9g06: Number of relevant climate change adaptation training programmes that integrate cultural heritage and/or guidance from traditional, local or Indigenous knowledge | This indicator measures the extent to which training and awareness raising regarding climate change adaptation integrates i) cultural heritage considerations where cultural heritage includes tangible, intangible, and natural heritage; ii) adaptive strategies and practices guided by traditional, local, or Indigenous knowledge. This indicator also serves to address the MOI indicators regarding capacity building and access. | A majority of training programmes and awareness raising initiatives regarding climate change adaptation, from the international to local levels, do not include considerations of cultural and natural heritage sites or of intangible heritage elements/practices. They neither include adaptive practices that are guided by traditional, local, or indigenous knowledge. Hence, this indicator aims to highlight and mobilize Parties to integrate cultural heritage considerations in all relevant training programmes at all levels as well as including adaptive practices guided by diverse knowledge systems including traditional, local, or Indigenous knowledge where applicable | Some data is available with UNESCO for climate change adaptation programmes, sustainability integration/awareness, education and training curricula https://whc.unesco.org/en/indicators2030/ and https://www.unesco.org/en/links and from Parties from training programmes organized through agencies, and programmes authorized to conduct such training using national, subnational, or local funds, or funds from multilateral or bilateral donors. Under the UNFCCC Convention, such data is likely in NAPs or other national documents (NDC, ADCOMs, BTRs, National Communications etc.), other sources include also IPCC, academic literature, grey literature and implementation reports. Data is likely found also in organisations that work directly on climate change adaptation such as NGOs, climate funds, research organisations, that either invest in or deliver such programs | Number |
| 9g07: Percentage of cultural heritage specific climate adaptation measures that engage with and are informed by local or Indigenous Peoples and their knowledge systems | This indicator measures the extent to which climate change adaptation strategies for cultural and natural heritage i) engage the participation of local communities or Indigenous Peoples; ii) are informed by local, traditional, or Indigenous knowledge  Hence this indicator aims to measure climate change adaptation measures specific to cultural or natural heritage that are guided/informed by local, traditional, or Indigenous knowledge systems as well as those that have engaged the participation of local communities and Indigenous Peoples. | Climate adaptation measures for cultural heritage would benefit from an increasing engagement with diverse knowledge systems and stakeholder groups (traditional, local or Indigenous) that can strengthen adaptation of all types of cultural heritage. The IPCC AR6 SYR found with high confidence that "meaningful participation and inclusive planning, informed by cultural values, Indigenous Knowledge, local knowledge, and scientific knowledge can help address adaptation gaps and avoid maladaptation”. | Data on adaptation measures that engage Indigenous peoples has been assessed in IPCC (AR5, AR6 and will be in AR7). Similarly, information and data sources under the UNFCCC such as NAPs or other national climate change documents (NDC, ADCOMs, BTRs, NDCs etc.), other sources such as academic literature, grey literature e.g. implementation reports.  Some data on adaptation measures that engage with local or Indigenous Peoples and their knowledge systems are available at UNESCO: Dive into intangible cultural heritage! and https://ich.unesco.org/en/ and https://www.unesco.org/en/links and https://whc.unesco.org/en and https://whc.unesco.org/en/indicators2030/ | Percentage |
| 9g08: Number of cultural heritage buildings and sites retrofitted with climate-resilient materials and/or technologies, including those guided by traditional, local, or Indigenous building practices | This indicator focuses specifically on climate adaptation of historic buildings and sites that are vulnerable to climate change impacts and hazards. It includes retrofitting with i) climate-resilient materials and ii) technologies iii) while also taking into consideration the traditional, local or Indigenous building practices that can guide such retrofitting where applicable. New construction that integrates adaptive practices guided by traditional, local, or Indigenous building practices are also included. | In addition to cultural heritage sites, historic buildings and building stock should be repurposed and reused, where appropriate, retrofitting to protect them from climate change related impacts and hazards. Such retrofitting can be based on (i) new/scientific climate resilient materials or (ii) new or existing technologies or (iii) guided by traditional, local or Indigenous building practices that already integrate solutions and knowledge responsive to the local geography. Such retrofitting may include new construction guided by traditional, local, or Indigenous building practices. | Data is available with Parties at the national, subnational, and local levels including authorities responsible for housing and urban development. Some data is available with UNESCO https://whc.unesco.org/en/ and https://whc.unesco.org/en/indicators2030/. Under the UNFCCC Convention, such data is likely in NAPs or other national documents (NDC, ADCOMs, BTRs, National Communications etc.), other sources include also IPCC, academic literature, grey literature and implementation reports. | Number |

# **Supplemental Table 2: List of potential indicators under other GGA targets determined to have relevance to GGA Target 9g Cultural heritage**

| **TARGET** | **INDICATOR NAME** |
| --- | --- |
| 9(d) Reducing climate impacts on ecosystems and biodiversity, and accelerating the use of ecosystem-based adaptation and nature-based solutions, including through their management, enhancement, restoration and conservation and the protection of terrestrial, inland water, mountain, marine and coastal ecosystems; | 9d01: Proportion of population with secured access to provisioning services from ecosystems that support adaptive capacity  9d03: Proportion of populations benefiting from nature- and biodiversity-based cultural services that enhance adaptive capacity  9d04: Area under restoration for enhancing ecosystem resilience and services  9d05: Extent of ecosystems that contribute to climate resilience covered by protected areas and other effective area-based conservation measures  9d06: Ecosystem resilience under climate change (measured by the Bioclimatic Ecosystem Resilience Index, BERI)  9d07: Threat status of ecosystems relevant for climate adaptation (Red List of Ecosystems)  9d08: Threat status of species relevant for climate adaptation (Red list index)  9d09: Extent of natural ecosystems relevant for climate adaptation |
| 9(e) Increasing the resilience of infrastructure and human settlements to climate change impacts to ensure basic and continuous essential services for all, and minimizing climate-related impacts on infrastructure and human settlements | 9e03: Number of Parties that include coverage of (i) critical thresholds, (ii) tipping points, and (iii) adaptation limits in National Adaptation Plans and national risk assessments  9e04: Number of planned relocation protocols for human settlements and infrastructural system to facilitate inclusive and adequately supported and managed local-scale relocation  9e06: Number of Parties that have established relevant standards or taxonomies designed to align public and private sector finance for Infrastructure & Human Settlement adaptation  9e07: Number of Parties that have national adaptation plans, policy instruments and/or strategies which include a consideration of the impact of temperature goal overshoot on the effectiveness of adaptation in relation to basic infrastructure |
| 9(f) Substantially reducing the adverse effects of climate change on poverty eradication and livelihoods, in particular by promoting the use of adaptive social protection measures for all | 9f01: Proportion of population living in multidimensional poverty in areas highly exposed to climate-related hazards  9f02: Proportion of population living below the international poverty line in areas highly exposed to climate-related hazards  9f03: Proportion of population living below the national poverty line in areas highly exposed to climate-related hazards |
| 10(c) Implementation: by 2030 all Parties have progressed in implementing their national adaptation plans, policies and strategies and, as a result, have reduced the social and economic impacts of the key climate hazards identified in the assessments referred to in paragraph 10(a) above | 10c03: Number of people who experienced direct social and economic impacts associated with climate-related hazards per 100,000 people  10c04: Direct economic loss associated with climate-related hazards as a proportion of gross domestic product  10c05: Costs of adaptation actions identified in adopted national adaptation plans, policy instruments, and planning processes and/or strategies  10c06 - Option 1: [Option 1] Amount of international public finance for climate adaptation provided, mobilized, and received for the implementation of national adaptation plans, policy instruments and planning processes and/or strategies per [time frame]  10c06 - Option 2: [Option 2] Amount of international public finance for climate adaptation provided, and received for the implementation of national adaptation plans, policy instruments and planning processes and/or strategies per [time frame]  10c06 - Option 3: [Option 3] Amount of international public finance for climate adaptation provided or mobilized by developed countries and received by developing countries for the implementation of national adaptation plans, policy instruments and planning processes and/or strategies per [time frame]  10c06 - Option 4: [Option 4] Amount of international public finance for climate adaptation provided by developed countries and received by developing countries for the implementation of national adaptation plans, policy instruments and planning processes and/or strategies per [time frame]  10c07: Annual adaptation finance expenditure  10c08 - option 1: [Option 1] Amount of private sector finance directed towards climate adaptation annually including private finance mobilized through public interventions  10c09 - option 1: [Option 1] Level of implementation of adaptation technology needs identified by developing countries, including needs expressed in Technology Needs Assessments (TNAs), NAPs, NDCs and other equivalent policy instruments, including their development and transfer from developed to developing countries  10c09 - option 2: [Option 2] Level of implementation of adaptation technology needs identified by Parties, including needs expressed in Technology Needs Assessments (TNAs), NAPs, NDCs and other equivalent policy instruments including their development and transfer  10c10: Number of Parties with institutional arrangements for the provision of regular training on climate change adaptation at the national and local level for governments and non-government organisations including community-based organisations  10c09 - option 1: [Option 1] Level of implementation of adaptation technology needs identified by developing countries, including needs expressed in Technology Needs Assessments (TNAs), NAPs, NDCs and other equivalent policy instruments, including their development and transfer from developed to developing countries  10c09 - option 2: [Option 2] Level of implementation of adaptation technology needs identified by Parties, including needs expressed in Technology Needs Assessments (TNAs), NAPs, NDCs and other equivalent policy instruments including their development and transfer  10c10: Number of Parties with institutional arrangements for the provision of regular training on climate change adaptation at the national and local level for governments and non-government organisations including community-based organisations  10c11: Extent of capacity-building interventions enhancing adaptive capacity of vulnerable communities/people |

# **Supplemental Table 3: List of potential adopted indicators under other GGA targets determined to have relevance to GGA Target 9g Cultural heritage**

| **Target** | **Indicator Examples** | **Heritage Relevance** |
| --- | --- | --- |
| Target 9(a) | Indicator 9a(c): “Proportion of critical water and sanitation infrastructure systems that are resilient to climate-related hazards under different warming scenarios, as appropriate for regions and contexts, including as an outcome of adaptation actions where applicable”  Indicator 9a(d): “Proportion of the total area of basins and cryosphere for which a climate adaptation plan has been developed and implemented on the basis of different warming scenarios, as appropriate for regions and contexts where applicable”  Indicator 9a(e): “Proportion of the population using safe and affordable potable water services that are climate-resilient, including as an outcome of adaptation actions where applicable”  Indicator 9a(g): “Extent of measures taken to improve and extend water, sanitation and hygiene services to populations disproportionately affected by climate change and to vulnerable groups relative to needs” | Indicator 9a(c): Encompasses traditional water infrastructure such as qanats, step wells, terraced irrigation systems, and traditional tanks that function simultaneously as water systems and cultural heritage.  Indicator 9a(d): Creates an opportunity for basin-level adaptation planning to recognize and strengthen Indigenous water management systems and sacred water sources, including springs, rivers, and wetlands embedded in cultural landscapes.  Indicator 9a(e): May include populations served by heritage water systems if Parties recognize traditional infrastructure as climate-resilient, although standard interpretation often prioritizes modern engineered systems.  Indicator 9a(g): Becomes relevant where Indigenous Peoples and traditional communities reliant on heritage water systems are recognized as vulnerable groups disproportionately affected by climate change. |
| Target 9(b) | Indicator 9b(a): “Proportion of area under management for food and agricultural production utilizing practices and technologies relevant to climate change adaptation”  Indicator 9b(b): “Extent of implementation of institutional frameworks for knowledge transfer, research and development, and extension services supporting climate change adaptation in the areas of food and agriculture relative to needs”  Indicator 9b(c): “Level of degraded areas that are under management for food and agricultural production, including as an outcome of adaptation actions where applicable” | Indicator 9b(a): Directly includes traditional agroecological practices such as intercropping, crop rotation, use of traditional seed varieties, agroforestry, pastoral transhumance, and customary fishing techniques, all of which constitute agricultural heritage.  Indicator 9b(b): Could capture transmission of traditional agricultural knowledge, although the emphasis on formal knowledge systems may marginalize community-based heritage practices.  Indicator 9b(c): Intersects with traditional land-management practices such as terracing, Indigenous grazing systems, and customary fire management used to restore degraded heritage landscapes. |
| Target 9(c) | Indicator 9c(d): “Proportion of the population vulnerable to climate change with access to mental health and psychosocial support”  Indicator 9c(g): “Coverage of essential health services that are supported by adaptation measures to ensure continuity during and following climate-related events”  Indicator 9c(h): “Proportion of health practitioners who have received capacity-building support pertaining to climate change adaptation and health” | Indicator 9c(d): Relevant to climate-induced cultural grief and psychological distress resulting from loss of heritage sites, disruption of cultural practices, or displacement from ancestral lands.  Indicator 9c(g): May encompass Indigenous and traditional health systems where these are recognized as essential services.  Indicator 9c(h): Could include training related to cultural dimensions of climate impacts, including the mental health consequences of heritage loss. |
| Target 9(d) | Indicator 9d(a): “Proportion of climate-resilient ecosystems that are providing services to populations that depend on them”  Indicator 9d(b): “Proportion of ecosystem areas with adaptation actions implemented towards enhanced resilience and services”  Indicator 9d(c): “Level of resilience of ecosystems, including as an outcome of adaptation actions where applicable”  Indicator 9d(f): “Level of adaptive capacity, resilience and vulnerability to climate impacts resulting from the implementation of ecosystem-based adaptation actions and nature-based solutions…” | Indicator 9d(a): Directly relevant to sacred natural sites, cultural landscapes, and Indigenous territories that provide ecological and cultural services simultaneously.  Indicator 9d(b): Encompasses adaptation strategies in biocultural landscapes maintained through traditional practices such as sacred grove management or customary marine tenure.  Indicator 9d(c): Relevant where ecosystem resilience and cultural continuity are interdependent.  Indicator 9d(f): Includes Indigenous-led conservation practices, traditional fire knowledge, and sacred natural site stewardship, which serve as both adaptation measures and cultural heritage expressions. |
| Target 9(e) | Indicator 9e(a): “Proportion of settlement upgrading programmes implemented that include climate change adaptation measures and maintain sustained engagement at the local level”  Indicator 9e(b): “Proportion of infrastructure and human settlements vulnerable to climate-related hazards and other extreme events relocated to a safer location” | Indicator 9e(a): Affects historic districts, vernacular settlements, and culturally significant neighborhoods where adaptation measures must consider cultural values and local knowledge.  Indicator 9e(b): Raises concerns where relocation threatens cultural continuity, community identity, or the future of heritage settlements. |
| Target 9(f) | Indicator 9f(a): “Level of population living in poverty, including as an outcome of adaptation actions where applicable”  Indicator 9f(b): “Proportion of the population in climate-vulnerable areas with access to social protection services”  Indicator 9f(c): “Level of social protection systems that consider climate risk management aspects and can respond to climate change impacts” | Indicator 9f(a): Relevant where climate impacts on heritage sites undermine heritage-based economies.  Indicator 9f(b): Intersects with communities whose livelihoods depend on heritage-based activities such as craft production, traditional subsistence practices, or cultural tourism.  Indicator 9f(c): Presents an opportunity to design protections for heritage-based livelihoods, though heritage is not explicitly referenced. |
| Target 10(b) | Indicator 10b(c): “Existence of national adaptation plans, policy instruments, planning processes and strategies that have been informed by traditional knowledge, knowledge of Indigenous Peoples and local knowledge systems” | Indicator 10b(c): Directly aligns with the knowledge foundations of intangible cultural heritage and complements Target 9(g)’s implementation indicators. |
| Target 10(c) | Indicator 10c(d): “Amount of finance for climate adaptation reported in line with chapters IV, V and VI, as relevant and as appropriate, of the annex to decision 18/CMA.1, disaggregated by the parameters listed in paragraphs 123, 125, 133 and 134, as applicable, of the same decision, which includes the amount of international public finance for climate adaptation provided by developed countries and received by developing countries for the implementation of national adaptation plans, policy instruments, and planning processes and/or strategies”  Indicator 10c(e): “Technology development and transfer for climate adaptation reported in line with chapters IV, V and VI, as relevant and as appropriate, of the annex to decision 18/CMA.1, disaggregated by the parameters listed in paragraphs 127, 136 and 138, as applicable, of the same decision, which includes technology development and transfer support for climate adaptation provided by developed countries and needed and received by developing countries for the implementation of national adaptation plans, policy instruments, and planning processes and/or strategies”  Indicator 10c(f): “ Capacity-building for climate adaptation reported in line with chapters IV, V and VI, as relevant and as appropriate, of the annex to decision 18/CMA.1, disaggregated by the parameters listed in paragraphs 129, 140 and 142, as applicable, of the same decision, which includes capacity-building support for climate adaptation provided by developed countries and needed and received by developing countries for the implementation of national adaptation plans, policy instruments, and planning processes and/or strategies” | Indicator 10c(d): Captures whether adaptation finance reaches heritage-related needs, although heritage is not explicitly named in reporting categories.  Indicator 10c(e): May include technologies essential for heritage preservation, such as climate monitoring tools or digital documentation systems.  Indicator 10c(f): May include training for heritage custodians, Indigenous communities, and institutions to implement heritage-sensitive adaptation. |
